# Serum beta2-microglobulin acts as a biomarker for severity and prognosis in glioma patients: a preliminary clinical study

**DOI:** 10.1186/s12885-024-12441-0

**Published:** 2024-06-06

**Authors:** Zhen-Yuan Liu, Feng Tang, Jing Wang, Jin-Zhou Yang, Xi Chen, Ze-Fen Wang, Zhi-Qiang Li

**Affiliations:** 1 Brain Glioma Center, Department of Neurosurgery, Zhongnan Hospital of Wuhan University, Wuhan, Hubei China; 2 Department of Physiology, Wuhan University School of Basic Medical Sciences, Wuhan, Hubei China; 3 Department of Clinical Laboratory, Nanjing Jiangning Hospital, Nanjing, Jiangsu China; 4 Hubei International Science and Technology Cooperation Base for Research and Clinical techniques for Brain Glioma Diagnosis and Treatment, Wuhan, Hubei China

**Keywords:** Glioma, Glioblastoma, Beta2-microglobulin, Prognosis, Biomarker

## Abstract

**Background:**

Gliomas are the deadliest malignant tumors of the adult central nervous system. We previously discovered that beta2-microglobulin (B2M) is abnormally upregulated in glioma tissues and that it exerts a range of oncogenic effects. Besides its tissue presence, serum B2M levels serve as biomarkers for various diseases. This study aimed to explore whether serum B2M levels can be used in the diagnosis and prognosis of gliomas.

**Methods:**

Medical records from 246 glioma patients were retrospectively analyzed. The relationship between preoperative serum B2M levels and clinicopathological features was examined. Kaplan-Meier analysis, alongside uni- and multivariate Cox regression, assessed the association between B2M levels, systemic inflammatory markers, and glioma patient prognosis. Receiver operating characteristic (ROC) curve analysis evaluated the diagnostic significance of these biomarkers specifically for glioblastoma (GBM).

**Results:**

Patients with malignant gliomas exhibited elevated preoperative serum B2M levels. Glioma patients with high serum B2M levels experienced shorter survival times. Multivariate Cox analysis determined the relationship between B2M levels (hazard ratio = 1.92, 95% confidence interval: 1.05–3.50, *P* = 0.034) and the overall survival of glioma patients. B2M demonstrated superior discriminatory power in distinguishing between GBM and non-GBM compared to inflammation indicators. Moreover, postoperative serum B2M levels were lower than preoperative levels in the majority of glioma patients.

**Conclusions:**

High preoperative serum B2M levels correlated with malignant glioma and a poor prognosis. Serum B2M shows promise as a novel biomarker for predicting patient prognosis and reflecting the therapeutic response.

**Supplementary Information:**

The online version contains supplementary material available at 10.1186/s12885-024-12441-0.

## Introduction

Diffuse glioma, the most prevalent malignant brain tumor in adults with high recurrence rate and poor prognosis, constitutes approximately 29.7% of all central nervous system (CNS) neoplasms [1]. The World Health Organization (WHO) integrated molecular and histological features of tumors in 2021 to establish standardized criteria for classifying and grading gliomas [2]. Adult gliomas are primarily classified into three categories: astrocytomas (IDH-mutant without 1p/19q co-deletion, grade 2, 3, 4), oligodendrogliomas (IDH-mutant with 1p/19q co-deletion, grade 2, 3), and glioblastomas (IDH wild-type, GBM, grade 4) [3]. IDH mutant gliomas have entered the era of molecular targeted therapy [4], while the standard treatment for GBM is still maximal safe surgical resection in combination with radiotherapy and temozolomide chemotherapy [5, 6]. Therefore, early and accurately distinguishing between GBM and IDH mutant gliomas is of great significance for the selection of individualized treatment strategies for glioma patients. Besides, how to monitor tumor recurrence and predict prognosis of glioma patients using convenient and reliable indicator is also important.

Beta2-microglobulin (B2M), a 119-amino acid small protein with a molecular weight of 11.8 kDa, is synthesized by nearly all nucleated cells [7, 8]. Its canonical function is to act as a subunit of major histocompatibility complex class I (MHC-I) molecules, reinforcing the structural integrity of MHC-I and facilitating the presentation of intracellular antigens to natural killer (NK) cells and CD8^+^ T cells [9–11]. In solid tumors, B2M exhibits tumorigenic effects independent of MHC-I. In several human cancer types, including prostate, breast, and lung cancer, upregulated B2M interacts with the hemochromatosis proteins, leading to epithelial-mesenchymal transition and bone metastasis in cancer cells [12]. In prostate cancer, B2M triggers cAMP-dependent PAK activity, thereby promoting cell proliferation, viability, and angiogenesis [13]. In human renal cell carcinoma, B2M stimulates cancer cell proliferation and suppresses apoptosis by activating the PI3K/AKT and extracellular signaling pathways [14]. A recent study revealed that B2M maintains the stem cell properties of glioma stem cells through activation of the PI3K/ AKT/MYC signaling pathway [15].

Since B2M is non-covalently linked to the α-chain of MHC-I and not directly attached to the cell membrane, it has the potential to detach from the membrane and form free B2M [16, 17]. Soluble B2M is widely present in bodily fluids, including cerebrospinal fluid, urine, and serum [8, 18]. Soluble B2M concentrations in body fluids generally remain stable under normal physiological conditions, whereas elevated B2M levels are associated with several diseases. Recent clinical studies have shown elevated serum B2M levels in patients with hematological conditions, including follicular lymphoma, non-Hodgkin lymphoma, Hodgkin lymphoma, Burkitt lymphoma, and diffuse large B-cell lymphoma [19–23]. Elevated B2M levels are associated with disease severity and unfavorable prognosis for patients. Except for lymphomas, patients with CNS diseases like brain injury, Alzheimer’s disease, and acute cerebral infarction also display an increase in serum B2M levels [24–26]. Circulating B2M has the capability to traverse the blood-brain barrier (BBB) and contribute to neuronal damage [27, 28]. However, despite the observed pro-tumor effects of B2M in glioma, the utility of serum B2M as a biomarker in distinguishing between gliomas and non-gliomas, or between GBM and IDH mutant gliomas remains uncertain. Moreover, whether serum B2M could be used for monitoring tumor recurrence and predicting prognosis of glioma patients are also unknown.

## Methods

### Study population

This study retrospectively collected and analyzed medical records of glioma patients diagnosed at Zhongnan Hospital at Wuhan University, and Nanjing Jiangning Hospital from January 2017 to December 2022. A total of 246 patients were included based on the following inclusion criteria: (1) age > 18 years; (2) histological confirmation of glioma based on WHO standards; (3) no prior radiotherapy or chemotherapy before surgery; (4) no hematological or inflammatory diseases, other malignancies, or severe liver and kidney dysfunction; (5) availability of clinical information and complete preoperative data, including blood cell counts and serum B2M, albumin, globulin, and fibrinogen levels; and (6) provision of informed consent. Institutional ethics committee approval was obtained (No. 2,019,048).

Histological tumor type diagnosis and immunohistochemical results (ATRX deletion, IDH mutation, Ki-67 expression, etc.) were available for all 246 patients who underwent surgical intervention. Among them, 225 patients underwent molecular testing for glioma cells (*IDH1/2, TERT, PTEN, P53* mutations; *MGMT* promoter methylation status; *EGFR* amplification; presence of chromosome 1p/19q co-deletion, etc.). The study analyzed the association between preoperative serum B2M levels and glioma grade, pathologic status, and patient prognosis in these patients.

Postoperative serum B2M levels were assessed in 146 out of 246 glioma patients. Additionally, serum B2M levels were measured before and after treatment in four patients who experienced postoperative glioma recurrence and underwent re-surgery. In these patients, we explored the relationship between changes in serum B2M levels and surgical interventions. A timeline was created to visually illustrate the study’s progress (Figure S1A).

### Data collection

Demographic and clinical data of patients with glioma, including sex, age, tumor location and size, primary symptoms, Karnofsky Performance Scale score, tumor grade, and pathological molecular characteristics, were systematically retrieved from the hospital’s electronic records. Patients’ peripheral blood was routinely collected within two days of admission as part of the standardized preoperative assessment for subsequent blood tests, liver and kidney function assessments, and coagulation function tests. Collected data comprised B2M, albumin, globulin, and fibrinogen levels, as well as neutrophil, lymphocyte, monocyte, and platelet count. Additionally, we calculated the following predictive indicators:

NLR = neutrophil count/ lymphocyte count

PLR = platelet count/lymphocyte count

LMR = lymphocyte count/monocyte count

AFR = albumin level/fibrinogen level

AGR = albumin level/globulin level

PNI = albumin level + lymphocyte count × 5

SII = platelet count × the NLR

SIRI = (neutrophil × monocyte) count/lymphocyte count.

### Statistical analysis

Statistical analysis was performed using IBM SPSS version 26.0 (IBM Corp., Armonk, NY) and GraphPad Prism 8.0 software (GraphPad Prism, San Diego California, USA). Normal distribution of variables was initially assessed using the Kolmogorov-Smirnov test. For normally distributed data, Student’s t-test was used; otherwise, the Mann-Whitney U test was employed. The chi-squared test was used to compare categorical variables. Optimal cutoff values for B2M and inflammatory markers were determined using X-Tile software (version 3.6.1, Yale University School of Medicine, USA). Receiver operating characteristic (ROC) curves were used to identify the optimal cut-off values for B2M and systemic inflammation indicators. Postoperative survival rates were evaluated using the Kaplan-Meier method and log-rank test. Uni- and multivariate Cox proportional hazards regression models were used to compute hazard ratios (HR) and corresponding 95% confidence intervals (CI), to determine the effects. Statistical significance was set at *P* < 0.05.

## Results

### Study population characteristics

Following the inclusion criteria, 246 glioma patients were enrolled in the study. Table 1 presents detailed demographic information. The cohort comprised 153 males (62.2%) and 93 females (37.8%), with a median age at diagnosis of 54 years (range: 23–80 years). Headaches and seizures are common symptoms in individuals with glioma. According to the 2021 WHO classification criteria, 42 patients (17.1%) were grade 2, 43 (17.5%) were grade 3, and 161 (65.4%) were grade 4. Specifically, 52 patients (21.1%) were diagnosed with astrocytoma, 43 (17.5%) with oligodendroglioma, and 151 (61.4%) with glioblastoma.

**Table 1:** Demographic characteristics of the patients with gliomas

Parameter	Median	Patient number (%)
Gender		
Male		153 (62.2)
Female		93 (37.8)
Age	54 (23–80)	
Tumor location		
Frontal		80 (33.2)
Temporal		45 (18.7)
Parietal		26 (10.8)
Others		90 (37.3)
Karnofsky Performance Scale	80 (50–100)	
Tumor size, cm^3^	9.5 (0.05–189)	
Symptoms at the diagnosis		
Epilepsy		39 (16.2)
Headache		93 (38.6)
Dizziness		33 (13.7)
Limb weakness		26 (10.8)
Others		50 (20.7)
Glioma grade		
2		42 (17.1)
3		43 (17.5)
4		161 (65.4)
Histological types		
Astrocytoma		52 (21.1)
Oligodendroglioma		43 (17.5)
Glioblastoma		151 (61.4)
Postoperative radio- and/or chemotherapy		
Yes		141 (57.3)
No		8 (3.3)
NA		97 (39.4)
β2-MG in µg/L	1540.6 (822.0−2817.9)	
NLR	2.32 (0.62–59.76)	
PLR	121.76 (39.64–872.00)	
LMR	3.50 (0.26–12.29)	
AFR	0.13 (0.05–0.30)	
AGR	1.50 (0.92–2.65)	
PNI	47.55 (29.4−65.15)	
SII	456.3 (100.1−13027.7)	
SIRI	1.09 (0.19–57.37)	

### Serum B2M levels correlate with the malignancy of gliomas

**Fig. 1:**
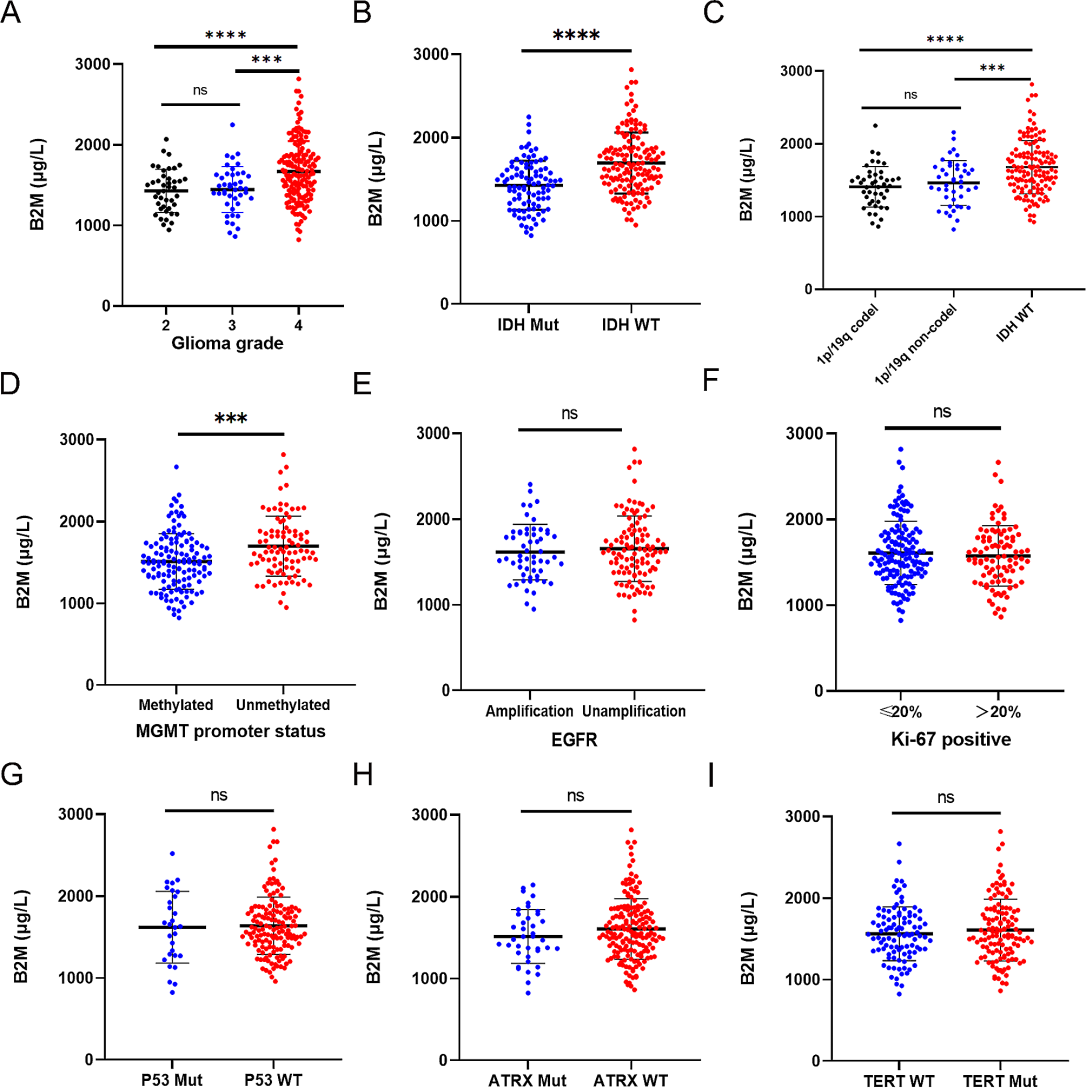
Differences in preoperative serum B2M levels of the various glioma subtypes in our cohort. **A-I.** The serum B2M levels were higher in high-grade gliomas (**A**), IDH wild-type gliomas (**B**-**C**), and MGMT promoter unmethylated gliomas (**D**), while independent of EGFR amplification, the rate of Ki-67 positivity, or mutations in *P53*, *ATRX*, and *TERT* (**E**-**I**). ***P < 0.001, ****P < 0.0001, ns means *P* > 0.05

Initially, we compared baseline serum B2M levels between glioma patients and non-malignant brain tumor patients, revealing higher levels in glioma patients (Figure S1B, Table S1). Subsequently, we investigated the association between serum B2M levels and glioma subtype. Results indicated a positive correlation between serum B2M levels and glioma grade, with higher levels observed in grade 4 gliomas compared to grades 2–3 (Fig. 1A). Regarding IDH-mutant gliomas, serum B2M levels were higher in IDH wild-type gliomas (Fig. 1B-C). In addition, glioma patients lacking *MGMT* promoter hypermethylation exhibited elevated B2M levels compared to those with hypermethylation (Fig. 1D). However, serum B2M levels in glioma patients appeared independent of other factors, including EGFR amplification, Ki-67 positivity rate, and mutations in *P53*, *ATRX*, and *TERT* (Fig. 1E-I). Thus, the findings indicate elevated serum B2M levels in malignant gliomas compared to non-malignant ones.

### Serum B2M serves as an independent prognostic factor for patients with glioma

**Fig. 2:**
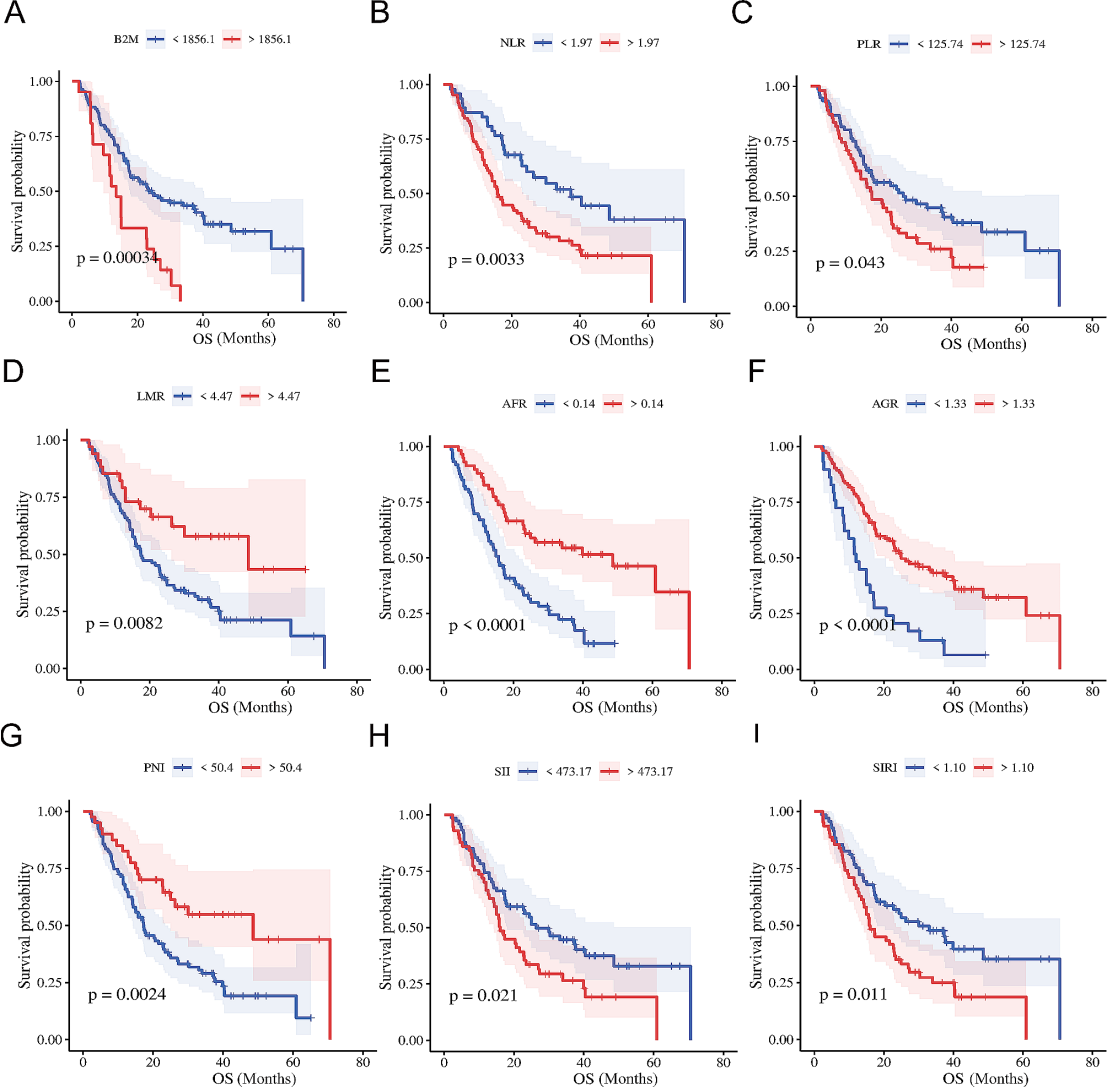
The association between serum B2M and inflammatory markers and prognosis in glioma patients. **A-I.** Glioma patients with higher B2M, NLR, PLR, SII, SIRI (**A**-**C** and **H**-**I**), or lower LMR, AFR, AGR, PNI (**D**-**G**) had poorer prognoses

Prior studies have shown associations between various serum biomarkers—NLR, LMR, AFR, and PNI—and glioma prognosis [29–32]. Using data from our dual-center glioma cohort, we assessed the prognostic value of these serum metrics, as well as B2M, in patients with glioma. The optimal cut-off values for each marker in our cohort are presented in Table S2. Patients with glioma exhibiting higher serum B2M levels experienced poorer prognoses (Fig. 2A). Our findings were consistent with previous research, showing that higher NLR, PLR, SII, and SIRI levels, or lower LMR, AFR, AGR, and PNI levels, were associated with poorer prognoses in glioma patients (Fig. 2B-I). Uni- and multivariate Cox regression analyses were performed to further evaluate the prognostic roles of these biomarkers in gliomas. While univariate analysis showed associations between all markers and glioma prognosis, only serum B2M and AFR emerged as independent prognostic factors in the multivariate analysis (Table 2). These findings indicate that the serum B2M level serves as an independent prognostic factor for patients with glioma.

**Table 2:** Univariate and multivariate analyses of OS in glioma cohorts

Variables	Univariate Analysis		Multivariate analysis	
	HR (95% CI)	*P* value	HR (95% CI)	*P* value
B2M (>1856.1 vs. ≤ 1856.1) (µg/L)	2.46 (1.48–4.11)	**0.001**	1.92 (1.05–3.50)	**0.034**
NLR (>1.97 vs. ≤ 1.97)	2.01 (1.25–3.23)	**0.004**	1.56 (0.79–3.10)	0.203
PLR (>125.74 vs. ≤ 125.74)	1.55 (1.01–2.38)	**0.045**	0.92 (0.51–1.65)	0.775
LMR (>4.47 vs. ≤ 4.47)	0.47 (0.27–0.83)	**0.01**	0.80 (0.40–1.58)	0.512
AFR (>0.14 vs. ≤ 0.14)	0.38 (0.24–0.61)	**<0.001**	0.49 (0.29–0.84)	**0.009**
AGR (>1.33 vs. ≤ 1.33)	0.44 (0.27–0.71)	**0.001**	0.76 (0.44–1.29)	0.305
PNI (>50.4 vs. ≤ 50.4)	0.46 (0.27–0.77)	**0.003**	0.67 (0.37–1.19)	0.170
SII (>473.17 vs. ≤ 473.17)	1.64 (1.07–2.51)	**0.022**	1.21 (0.64–2.29)	0.557
SIRI (>1.10 vs. ≤ 1.10)	1.73 (1.13–2.66)	**0.012**	0.80 (0.42–1.51)	0.486

Bold indicates statistical significance

### Serum B2M exhibits moderate sensitivity in diagnosing GBM

**Fig. 3:**
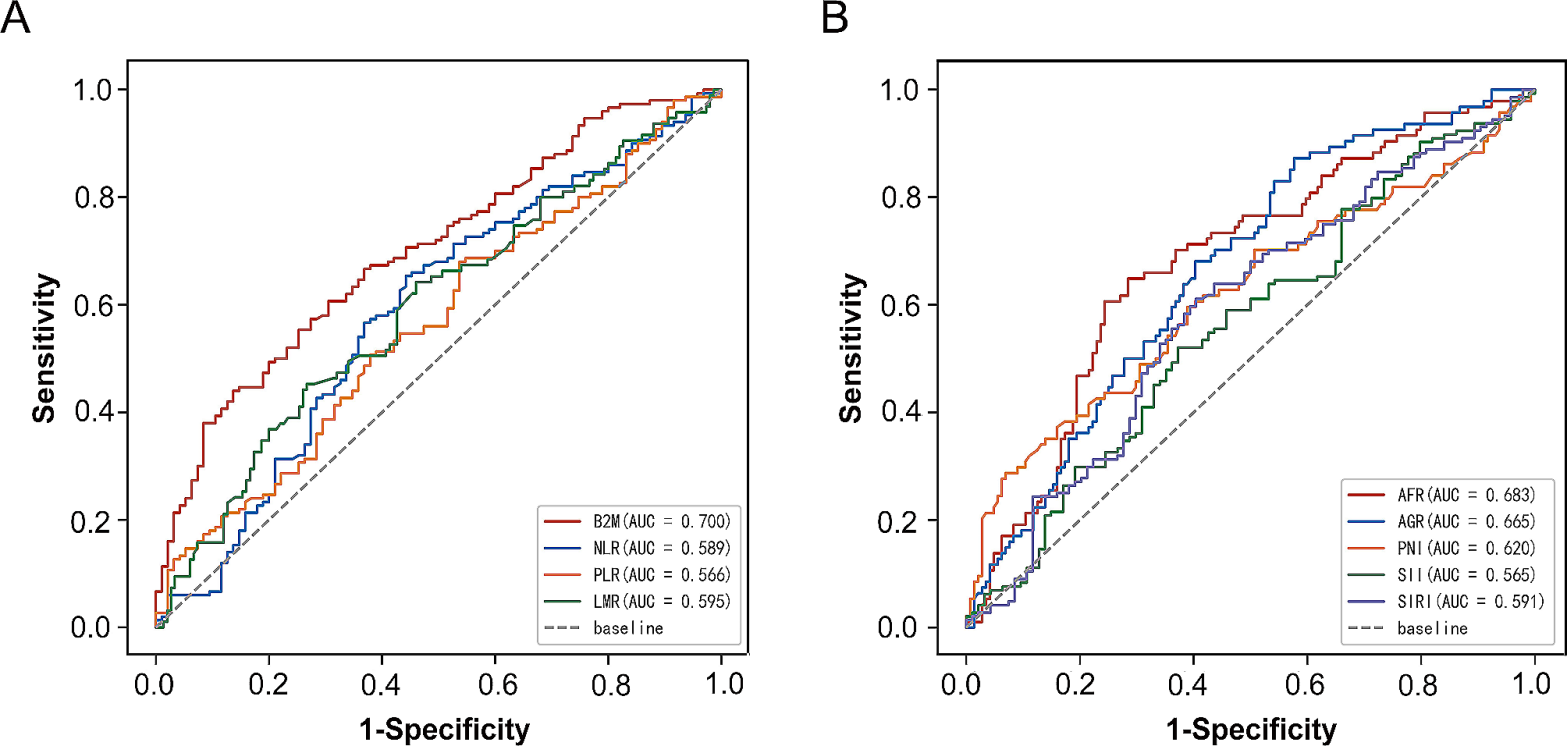
Diagnostic efficacy of B2M and serum inflammatory markers in patients with GBM.

Distinguishing between GBM and non-GBM patients prior to surgery is crucial due to different treatment strategies. We noted that the levels of most serum biomarkers in the two types of gliomas differed (Table 3), indicating that these biomarkers may be useful in differential diagnosis. We performed ROC analysis and the corresponding area under the curve (AUC) values are presented in Table 4. Three markers exhibited AUC values > 0.65 when patients with GBM were tested against non-GBM patients (Fig. 3): the AUC was 0.709 (0.643–0.774) for B2M, 0.683 (0.613–0.752) for AFR, and 0.665 (0.596–0.734) for AGR. Serum B2M levels exhibited the highest accuracy in predicting GBM. Thus, the serum B2M level serves as a moderately sensitive marker for diagnosing GBM.

**Table 3:** Parameter comparisons between the non-GBM and GBM groups

Parameter	Non-GBM (*n* = 95)	GBM (*n* = 151)	*P* value
β2-MG in µg/L	1454.8 (822.0−2249.1)	1637.3 (923.7−2817.9)	**<0.0001**
NLR	2.08 (0.69−19.00)	2.47 (0.62–59.76)	**0.02**
PLR	117.86 (49.05–362.50)	127.18 (39.64–872.00)	0.084
LMR	3.75 (0.83–8.51)	3.33 (0.26–12.29)	**0.012**
AFR	0.151 (0.073–0.299)	0.124 (0.053–0.256)	**<0.0001**
AGR	1.61 (1.13–2.65)	1.43 (0.92–2.63)	**<0.0001**
PNI	48.65 (37.60−59.65)	46.95 (29.40−65.15)	**0.002**
SII	424.33 (106.26–4218.00)	497.04 (100.10−13027.68)	0.089
SIRI	0.93 (0.19–22.80)	1.15 (0.24–57.37)	**0.026**

Bold indicates statistical significance

**Table 4:** Diagnostic value of parameters for distinguishing non-GBM from GBM

Parameter	Cut-off value	AUC (95% CI)
β2-MG in µg/L	1731.6	0.709 (0.643–0.774)
NLR	2.131	0.595 (0.520–0.669)
PLR	105.95	0.564 (0.490–0.638)
LMR	4.03	0.603 (0.529–0.677)
AFR	0.139	0.683 (0.613–0.752)
AGR	1.364	0.665 (0.596–0.734)
PNI	52.5	0.620 (0.545–0.696)
SII	486.38	0.565 (0.490–0.640)
SIRI	1.037	0.591 (0.516–0.665)

### Serum B2M holds potential as an indicator of treatment response in glioma patients

**Fig. 4:**
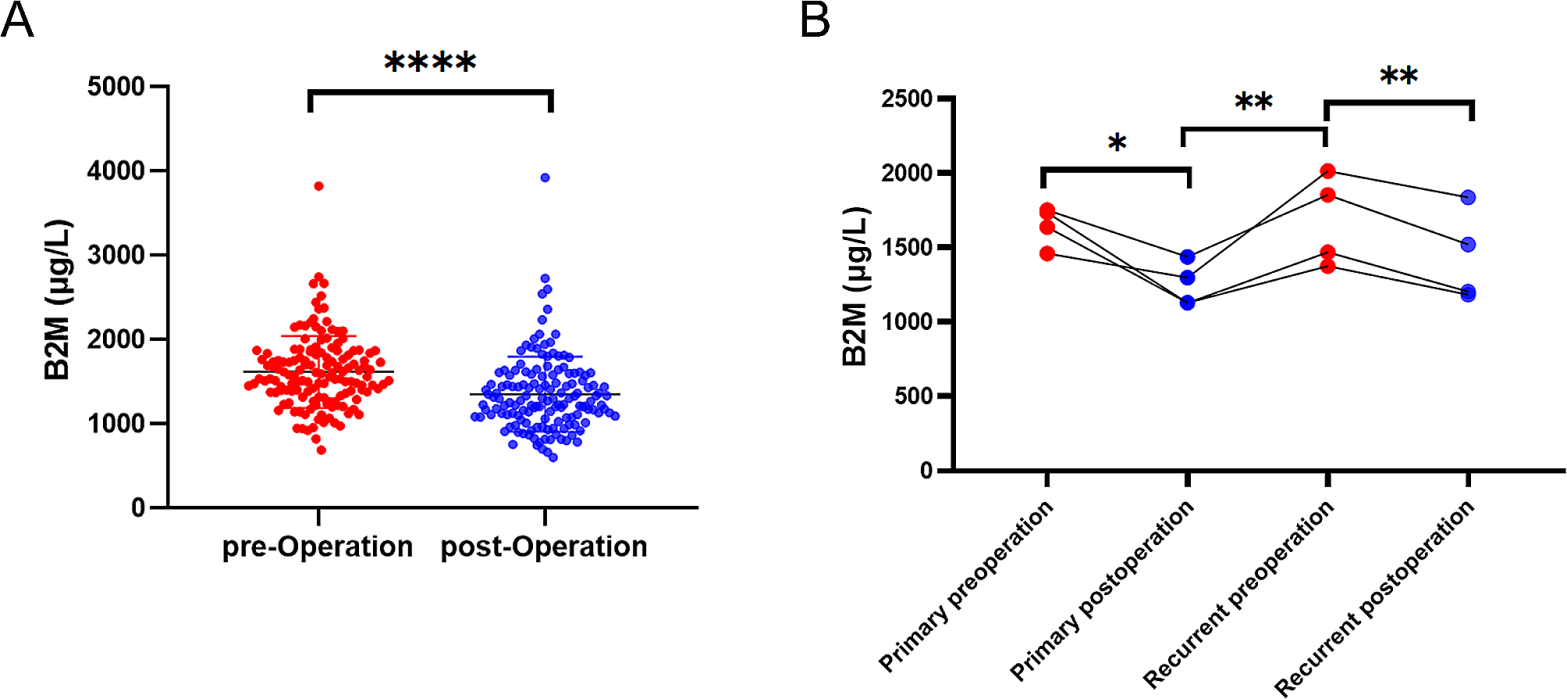
Differences in serum B2M levels in patients with glioma before and after surgical treatment. **A**, Changes in pre- and postoperative serum B2M levels in patients with primary gliomas. **B**, Changes in pre- and postoperative serum B2M levels between two operations in four patients with recurrent gliomas. *P < 0.05, **P < 0.01, ****P < 0.0001, paired t-test

We investigated the correlation between serum B2M levels and treatment response in glioma patients by collecting pre- and postoperative B2M data. Among these patients, 146 had both pre- and postoperative B2M data. The majority of patients exhibited a notable decrease in postoperative B2M levels compared to their preoperative levels (Fig. 4A). Interestingly, serum B2M levels decreased after the initial tumor surgery, rose after recurrence, and then declined again after the second surgery (Fig. 4B). The variations in serum B2M levels among patients with glioma aligned with their treatment, indicating the potential of B2M as a promising biomarker for assessing treatment response in patients with glioma.

## Discussion

The identification of circulating biomarkers suitable for population screening, early diagnosis, and tumor subclassification has been a prominent objective of oncology research, yielding significant advancements [33]. Unlike hepatocellular carcinoma and breast cancer, patients with glioma lack established serum biomarkers for clinical management [34]. B2M, a protein implicated in the initiation and progression of human tumors, has emerged as a promising candidate for cancer immunotherapy [7]. In a previous study, we observed a positive correlation between tissue B2M levels and glioma grade; patients with higher B2M levels had a poorer prognosis [35]. Subsequent bioinformatics analysis indicated that B2M in glioma cells may facilitate tumor development by modulating immune responses within the glioma microenvironment [36]. Recent studies have emphasized the critical role of B2M in maintaining stem cell characteristics of GBM stem cells. Furthermore, B2M activated the PI3K/AKT/MYC signaling pathway in glioma cells, resulting in TGF-β secretion and the polarization of tumor-associated macrophages into the M2 phenotype, promoting glioma progression [15, 37]. Nevertheless, despite the recognized role of B2M in glioma tumorigenesis and its acknowledgment as a serum biomarker for diverse diseases, it remained uncertain whether serum B2M levels in patients with glioma could serve as a biomarker for disease severity or prognosis. This study revealed that patients with malignant glioma phenotypes exhibited high serum B2M levels, which independently constituted a risk factor for unfavorable prognosis. B2M demonstrated better diagnostic efficacy for identifying GBM compared to other serum markers. Importantly, fluctuations in serum B2M levels correlated with glioma patients’ responses to treatment, potentially offering valuable insights for future clinical management [38].

Serum B2M, a small molecule capable of crossing the BBB, is involved in various CNS diseases. In aging mouse models, intravenously administered B2M accumulated in brain tissue, leading to memory and cognitive impairment through neurogenesis inhibition [39]. In a rat stroke model, clamping of the middle cerebral artery elevated brain B2M levels, triggering neuroinflammation via activation of the neuronal inflammasome NLRP3. Knockdown of serum B2M significantly improved the post-ischemic neuroinflammatory response [40]. Similarly, in Alzheimer’s disease, B2M co-aggregated with Aβ protein, contributing to neuronal degradation. Antibodies targeting B2M markedly enhanced neuronal survival [27]. These findings suggest that B2M generated by CNS disorders exerts a pathogenic effect and can translocate from the CNS to the serum through the BBB. In this study, we revealed a positive correlation between increased serum B2M levels and malignant gliomas; however, the exact molecular mechanisms remain unclear. We did not observe a correlation between B2M levels and the Ki-67 positivity rate of glioma cells. Furthermore, our bioinformatics analyses, along with others, indicate that B2M mainly influences anti-tumor immune suppression by affecting the glioma immune microenvironment [35, 36]. Based on this information, we hypothesize that, as a secreted protein, B2M may function similarly to Chitinase-3-like 1 protein (CHI3L1) [41], predominantly modulating the glioma immune microenvironment rather than the tumor cells per se. However, these hypotheses require further validation.

The inflammatory response significantly influences tumor progression and metastasis [42–44]. Certain common blood inflammatory markers are implicated in the prognosis of patients with diverse tumors. In glioma, several inflammatory markers, such as NLR, PLR, LMR, and PNI, have been identified to evaluate glioma grade and predict patient prognosis [45–50]. However, these indicators are still controversial. In this study, we incorporated these markers and conducted a comparative analysis of the serum B2M levels. Consistent with previous research, our cohort showed that patients with glioma having higher NLR, PLR, SII, and SIRI, or lower LMR, AFR, AGR, and PNI had a poorer prognosis. Importantly, the albumin-to-fibrinogen ratio (AFR) exhibited a remarkable capacity to determine the prognosis of patients with glioma and, similar to serum B2M, was an independent prognostic risk factor for these patients. Furthermore, the AFR had a high diagnostic value for GBM, second only to serum B2M levels. Clinical studies have explored the association between the AFR and patient prognosis in several diseases, including heart failure and percutaneous coronary intervention [51, 52]. However, in gliomas, this biomarker appears to be underexplored and warrants further examination in future research.

This study has certain limitations. First, our cohort comprised clinical data from 246 patients with gliomas across two hospitals, potentially leading to an insufficient sample size. Second, we did not collect data on the changes in B2M levels before and after postoperative radiotherapy and chemotherapy in patients with glioma, which reduces our confidence in the ability of serum B2M levels to reflect the response of patients with glioma to treatment.

## Conclusions

Our present findings suggest that preoperative serum B2M levels in patients with glioma are associated with disease severity and prognosis, potentially serving as a biomarker for the monitoring of the treatment response.

### Electronic supplementary material

Below is the link to the electronic supplementary material.

Supplementary Material 1

## Data Availability

Data used to support the findings of this study are available from the corresponding author upon request.
