# Supplementary material for: Serum beta2-microglobulin acts as a biomarker for severity and prognosis in glioma patients: a preliminary clinical study
Source: BMC Cancer. 2024 Jun 6;24:692. doi: 10.1186/s12885-024-12441-0 (PMC11155066; doi:10.1186/s12885-024-12441-0)
Supplement: Supplementary file 1 — Supplementary Material 1 [file 12885_2024_12441_MOESM1_ESM.docx]

**Serum Beta2-Microglobulin Acts as a Biomarker for Severity and Prognosis in Glioma Patients: A Preliminary Clinical Study**

Zhen-Yuan Liu^1*^, Feng Tang^1*^, Jing Wang^3^，Jin-Zhou Yang^1^, Xi Chen^1^, Ze-Fen Wang^2#^, Zhi-Qiang Li^1,4#^

1, Brain Glioma Center & Department of Neurosurgery, Zhongnan Hospital of Wuhan University, Wuhan, Hubei, China

2, Department of Physiology, Wuhan University School of Basic Medical Sciences, Wuhan, Hubei, China

3, Department of Clinical Laboratory, Nanjing Jiangning Hospital, Nanjing, Jiangsu, China

4, Hubei International Science and Technology Cooperation Base for Research and

Clinical techniques for Brain Glioma Diagnosis and Treatment, Wuhan, Hubei, China

* These authors have contributed equally to this work

# Corresponding authors：

Zhi-Qiang Li

Brain Glioma Center & Department of Neurosurgery, Zhongnan Hospital of Wuhan University, Wuhan, Hubei, China

Hubei International Science and Technology Cooperation Base for Research and

Clinical techniques for Brain Glioma Diagnosis and Treatment, Hubei, China

[lizhiqiang@whu.edu.cn](mailto:lizhiqiang@whu.edu.cn)

Ze-Fen Wang

Department of Physiology, Wuhan University School of Basic Medical Sciences, Wuhan, Hubei, China

[wangzf@whu.edu.cn](mailto:wangzf@whu.edu.cn；lizhiqiang@whu.edu.cn)


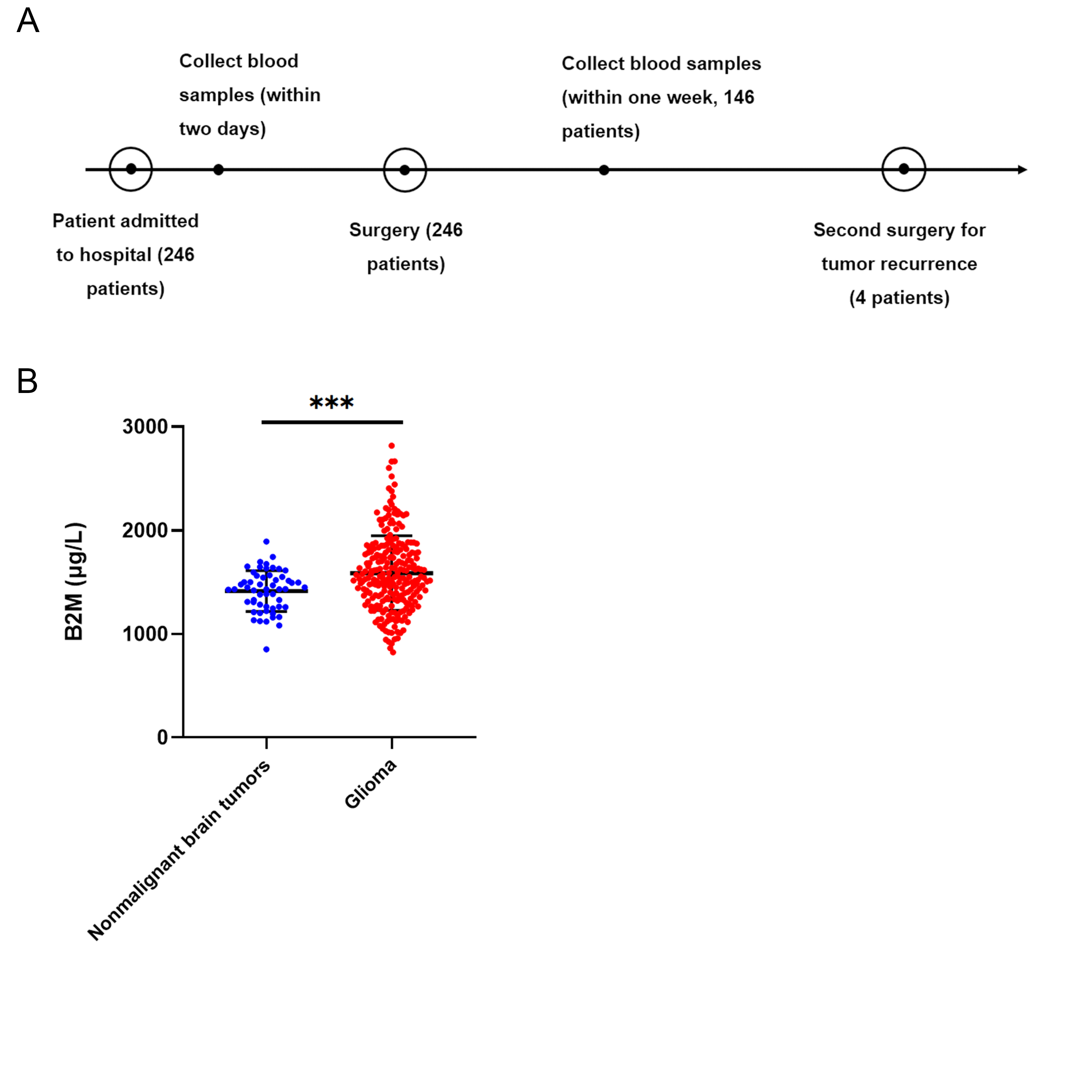


Figure S1. A, The study timeline. B, Patients with gliomas exhibit elevated serum B2M levels compared to those with non-malignant brain tumors.

| **Parameter** | **Median** | **Patient number (%)** | |
| --- | --- | --- | --- |
| Gender |  |  |  |
| Male |  | 15 (27.3) |  |
| Female |  | 40 (72.7) |  |
| Age | 45 (20-70) |  |  |
| Tumor type |  |  |  |
| Meningioma |  | 37 (67.3) |  |
| Pituitary tumor |  | 11 (20.0) |  |
| Craniopharyngioma |  | 7 (12.7) |  |

Supplementary Table 1. Demographic characteristics of the patients with non-malignant brain tumors.

Supplementary Table 2. Optimal cut-off values for each serum biomarker.

| **Parameter** | **Cut-off** |
| --- | --- |
| β2-MG in μg/L | 1856.1 |
| NLR | 1.97 |
| PLR | 125.74 |
| LMR | 4.47 |
| AFR | 0.14 |
| AGR | 1.33 |
| PNI | 50.4 |
| SII | 473.17 |
| SIRI | 1.10 |
